# Cancer Stem Cells: Significance in Origin, Pathogenesis and Treatment of Glioblastoma

**DOI:** 10.3390/cells10030621

**Published:** 2021-03-11

**Authors:** Karina Biserova, Arvids Jakovlevs, Romans Uljanovs, Ilze Strumfa

**Affiliations:** 1 Faculty of Residency, Riga Stradins University, 16 Dzirciema Street, LV-1007 Riga, Latvia; 2 Department of Pathology, Riga Stradins University, 16 Dzirciema Street, LV-1007 Riga, Latvia; Arvids.Jakovlevs@rsu.lv (A.J.); Romans.Uljanovs@rsu.lv (R.U.); Ilze.Strumfa@rsu.lv (I.S.)

**Keywords:** glioblastoma, glioma stem cells, glioma, cancer, cancer stem cells

## Abstract

Cancer stem cells (CSCs), known also as tumor-initiating cells, are quiescent, pluripotent, self-renewing neoplastic cells that were first identified in hematologic tumors and soon after in solid malignancies. CSCs have attracted remarkable research interest due to their role in tumor resistance to chemotherapy and radiation treatment as well as recurrence. Extensive research has been devoted to the role of CSCs in glioblastoma multiforme (GBM), the most common primary brain tumor in adults, which is characterized by a dismal prognosis because of its aggressive course and poor response to treatment. The aim of the current paper is to provide an overview of current knowledge on the role of cancer stem cells in the pathogenesis and treatment resistance of glioblastoma. The six regulatory mechanisms of glioma stem cells (GSCs)—tumor microenvironment, niche concept, metabolism, immunity, genetics, and epigenetics—are reviewed. The molecular markers used to identify GSCs are described. The role of GSCs in the treatment resistance of glioblastoma is reviewed, along with future treatment options targeting GSCs. Stem cells of glioblastoma thus represent both a driving mechanism of major treatment difficulties and a possible target for more effective future approaches.

## 1. Introduction

Cancer has attracted major research interest. Despite the tremendous progress in screening and treatment options, nowadays, malignant tumors rank as the leading cause of death. The growing input of cancer into the global death rate can be partially explained by the decreased mortality rates due to coronary heart disease and stroke [1]. Nevertheless, the cancer burden remains high, reaching an estimated 19.3 million new cases and 10 million cancer-induced death cases worldwide in 2020 [1]. In addition, the pathogenesis of cancer still remains incompletely understood.

Tumors of the central nervous system (CNS) do not rank among the most frequent neoplasms. Primary CNS tumors account only for 4% of all newly diagnosed cancer cases in the United States of America [2]. However, some of these neoplasms represent a major challenge for healthcare because of their dismal prognosis and poor response to treatment. In adults, glioblastoma is an example of an unsolved problem in neuro-oncology. CNS tumors are the most frequent cause of oncologic mortality in the 0–19 year age group and the second leading cause of oncological death in patients aged 20–39 years.

Regarding tumors of the brain, spinal cord, and surrounding tissues, more than 100 entities are recognized in the most recent classification of tumors of the central nervous system issued by World Health Organization in 2016 [2]. Glial tumors predominate in this spectrum, representing almost 90% of all CNS malignancies, and glioblastoma represents the most frequently diagnosed (69%) glioma [2,3,4]. Glioblastoma alone accounts for 50.8% of all malignant tumors of the central nervous system [2].

In a large study based on 315,184 cases of CNS tumors in the database of the Surveillance, Epidemiology, and End Results (SEER) program, the overall incidence rate for glioblastoma in 2000–2017 was characterized as stable [2]. In contrast, Philips et al. reported a growing incidence of glioblastoma in England from 1995 to 2015, Ho et al. described an increasing incidence rate of gliomas along with an enlarging proportion of glioblastoma diagnoses in the Netherlands from 1989 to 2010, and Swedish scientists emphasized that epidemiologic estimates regarding the incidence of glioblastoma are controversial and biased [4,5,6]. The age-standardized incidence rate of glioblastoma in England was 2.39 in 1995 and 5.02 in 2015 [5].

The prognosis of glioblastoma remains dismal despite the best available treatment. Nowadays, the gold standard of glioblastoma care includes surgical resection, followed by radiotherapy combined with concomitant or adjuvant chemotherapy with an alkylating agent, temozolomide, which induce single- and double-strand DNA breaks [7,8,9]. This protocol results in a median survival time of 14–18 months, with few reports on longer overall survival periods reaching 20.9 months [4,9,10]. Five-year survival is reached by less than 5% of patients [4,9]. Although these numbers are unfavorable, significantly lower survival rates have been reported in some studies, e.g., 7.9 months [11]. Worse outcomes can be attributed to the incomplete availability of treatment, as shown by Ho et al. [4]. Nevertheless, reports on inferior survival rates reflect true problems in glioblastoma care.

The clinical course of glioblastoma is characterized by almost inevitable recurrence within the first year of treatment and marked resistance to chemo- and radiotherapy. Resistance and recurrence are features classically associated with cancer stem cells—a minor population of quiescent, pluripotent, and self-renewing cancer cells.

The aim of the current article is to review the current knowledge on glioma stem cells (GSCs), including their historical background, definition, the six main regulatory mechanisms, and their role in glioblastoma multiforme (GBM) resistance to chemotherapy and radiation treatment, as well as future treatment options targeting GSCs.

## 2. A Glance at the Core Research on the Origin and Development of Glioblastoma

The first cases of glioblastoma were recognized at the end of the 19th century. As stated in a recent historical review by Stoyanov and Dzhenkov, the first gross reports, based on autopsy findings, were provided by Burns (1800) and Abernety (1804) [12]. Neither the tissue structure nor the cells of origin were known then; however, infiltrative growth lacking a clear border with healthy brain tissue was evident, suggesting malignant behavior. Rudolf Virchow described the light microscopy findings for malignant brain tumors (1865) and coined the term “glioma” [12].

Following the first descriptions, questions about the origin of gliomas arose. Cases described by Hallervorden in 1930 seemed to suggest a pathogenetic association between multiple sclerosis and glioblastoma. Another hypothesis was the possibility that glial tumors arise from encephalitis, presumably because of cases that supported this idea in 1938 [12]. In 1932, Russell described “intranuclear inclusion bodies”, stating that they can be associated with glioma etiology [12]. A theory related to embryonal neurogenesis and “embryonal mother cells” was proposed by H. Ribbert. Thus, the concept of glioblastoma as a glial tumor was not straightforward, although Rudolf Virchow classified glioblastoma as a glioma, suggesting an etiologic association with glial cells discovered by himself [12]. Bailey and Cushing created the basis of the classification of gliomas. However, they believed that the most atypical tumors, designated as spongioblastoma multiforme, were not of glial origin. This assumption was based on the marked cellular anaplasia. Scherer recognized the link between GBM and astrocytic tumors by showing that some astrocytomas can develop into GBM. Thus, the concept of secondary glioblastoma, arising from pre-existing astrocytoma, was defined [12]. In contrast, de novo glioblastomas, lacking the precursor lesion of lower-grade astrocytic tumors, were classified as primary glioblastomas. Nowadays, the concept of primary versus secondary glioblastoma has been supplemented by the mutational status of the isocitrate dehydrogenase gene *IDH* [12].

When glial features became evident in glioblastomas and astrocytomas, neoplastic transformation of mature, fully differentiated glial cells was considered to be the source of these tumors. This theory was supported by experimental data showing malignant transformation of astrocytes by ectopic introduction of oncogenes, for instance, *H-ras* and *hTERT*. In addition, glial cells are known to be able to proliferate under different pathological conditions [13,14].

More recently, neural stem cells and adult neurogenesis in neurogenic niches were discovered [15,16]. The presence of cells retaining the ability to proliferate in the adult brain suggested that glioblastomas might develop from neural stem cells [12]. This conversion has been shown experimentally. Thus, Lee et al. immortalized human fetal neural stem cells by *v-myc* and then induced malignant transformation using *H-ras* for oncogenic stimulation. Oligodendrocytes derived from the *v-myc*-expressing parent neural stem cells did not undergo malignant change after oncogenic stimulation by *H-ras*. Hence, the authors concluded that neural stem cells are more susceptible to neoplastic conversion than differentiated cells [13]. The malignant change might be driven by DNA replication stress, induced by the transcription of very long neural genes. The replication stress and development of R-loops can result in DNA double-strand breaks at specific chromosomal sites [17]. However, controversies exist. It has been shown in mouse models upon transduction by oncogenic lentiviral vectors that even highly differentiated brain cells, e.g., astrocytes and neurons, are able to undergo dedifferentiation resulting in GBM [14].

The history of glioblastoma research, from reports of the first cases to now, is astonishing. Despite the remarkable progress made over two centuries, questions still remain without complete and unambiguous answers. Glioblastoma represents the nature of cancer—a complex, dynamic process, involving a tremendous number of molecular mechanisms, signaling cascades, and cells.

## 3. Concept of Cancer Stem Cells and Glioma Stem Cells

The concept of cancer stem cells initially paralleled the hypothesis of normal tissue stem cells. Stem cells are self-renewing cells that maintain a capacity to proliferate, generating new stem cells and daughter cells that undergo differentiation and replenish the pool of functional cells. Stem cells are pluripotent—namely, they can give rise to different lineages of daughter cells. For instance, neural stem cell can generate cells that undergo differentiation into neurons, astrocytes, and oligodendrocytes.

Similarly, the traditional theory of cancer stem cells defines cancer stem cells (CSCs) as a minor subpopulation of self-renewing malignant cells that maintain a low but steady level of unlimited proliferation. Unlimited proliferation maintains the tumor, but rapid growth is dependent on the fast-dividing progeny of CSCs. The low mitotic activity of CSCs protects them from treatment approaches that are directed against actively dividing cells. Thus, CSCs can survive treatment and give rise to recurrences.

In experimental animal studies, CSCs were shown to behave like tumor-initiating cells. When these cells are introduced into the animal, they give rise to tumor growth, recapitulating the whole heterogeneity of neoplastic tissues. Nevertheless, the close association with tumor-initiating cells in animal models is not synonymous with CSCs as the cells of cancer origin [18].

The low proliferative activity, low abundance within the tumor, treatment resistance, and association between CSCs and tumor recurrence are accepted as the general features of cancer stem cells. These features have been demonstrated in glial stem cells (GSCs) in glioblastoma. GSCs are able to induce tumors in immunocompromised mice, recapitulating the heterogeneity and complexity of the initial tumor [19]. Further, in a mouse model, Chen et al. showed that a subset of tumor cells is the source of glioblastoma recurrence after temozolomide treatment; these cells express the stemness marker nestin and exhibit low Ki-67 proliferative activity [20]. GSC chemoresistance has been show in cell cultures as well, with escape from apoptosis suggested as the underlying mechanism [18]. The radio resistance of GBM has also been shown to be associated with GSCs expressing the stem cell marker CD133 [21].

The concept of CSCs does not necessarily imply that these cells are precursors of human tumors. However, the previously noted hypothesis assuming malignant change in neural stem cells as being the source of glioblastoma also proposes that GSCs develop during this process and further give rise to the development of GBM. This hypothesis proposes strictly hierarchical, unidirectional cellular proliferation. Considering the heterogeneity of GBM, an alternative dynamic hypothesis explains the origin of GSCs as being from non-stem tumor cells by dedifferentiation [20,21].

Glioblastomas are classified into molecular subtypes based on their gene expression profiles or immunohistochemistry [11,22,23]. Classically, four subtypes have been defined: proneural, neural, classical, and mesenchymal GBM. Interestingly, GSCs also can be classified into molecular subtypes. Mesenchymal and proneural GSCs have been reported, closely paralleling the dominant immunohistochemical subtypes of GBM [11]. In addition, primary proneural GBM can recur as mesenchymal GBM. The subtype switch reflects general feature of tumors and can be explained either by the occurrence of a molecular switch in proneural GSCs, leading to transformation into a mesenchymal profile, or by better survival of mesenchymal GSCs that were present in the primary proneural GBM [24,25].

## 4. Regulatory Mechanisms Influencing Glioma Stem Cells

**Figure 1:**
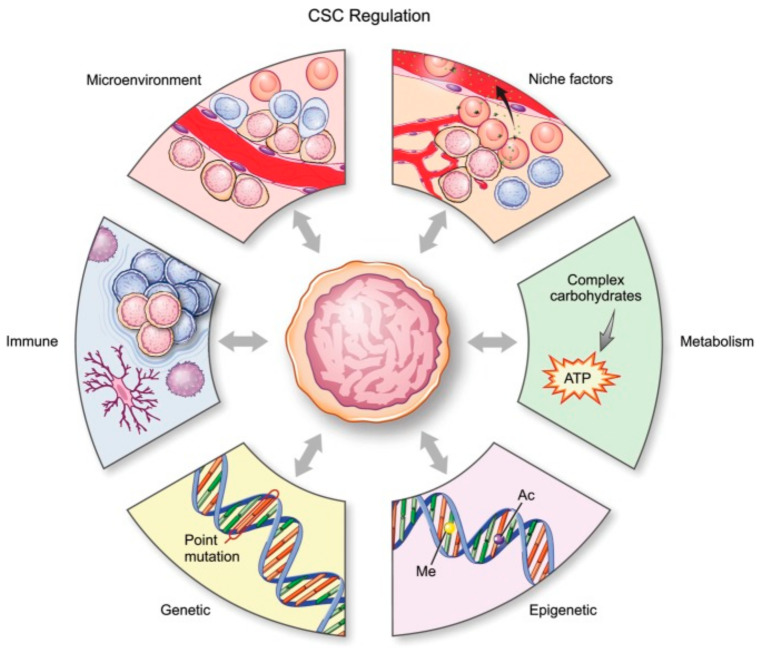
Schematic representation of all processes affecting glial stem cells (GSCs). [17]. CSC, cancer stem cell. License of this figure provided at https://creativecommons.org/licenses/by-nc/4.0/ (accessed on 19 February 2021). Changes made: figure description.

There are six main mechanisms (Figure 1) by which CSCs in glioma are regulated: genetic, epigenetic, and metabolic alterations, the tumor microenvironment, so-called niche qualities, and the immune system [18].

### 4.1. Tumor Microenvironment

The tumor microenvironment (TME) consists of all non-malignant elements present in the tumor that maintain, support, or hinder tumor evolution [26]. Stem cells can interact with the tumor microenvironment, thus promoting tumor growth, and the microenvironment, e.g., hypoxic conditions, can contribute to the generation of stem cells.

In glioblastoma, TME comprises glioma-associated microglia/macrophages, monocytes, dendritic cells, tumor-associated neutrophils, myeloid-derived suppressor cells, and normal and reactive astrocytes and pericytes [26,27,28,29,30,31,32]. However, the closest and most important interactions occur between the endothelium and GSCs via various molecular messengers and pathways, such as nitric oxide NO, cyclic guanosine monophosphate cGMP, and, most importantly, Notch signaling [33,34,35,36].

**Figure 2:**
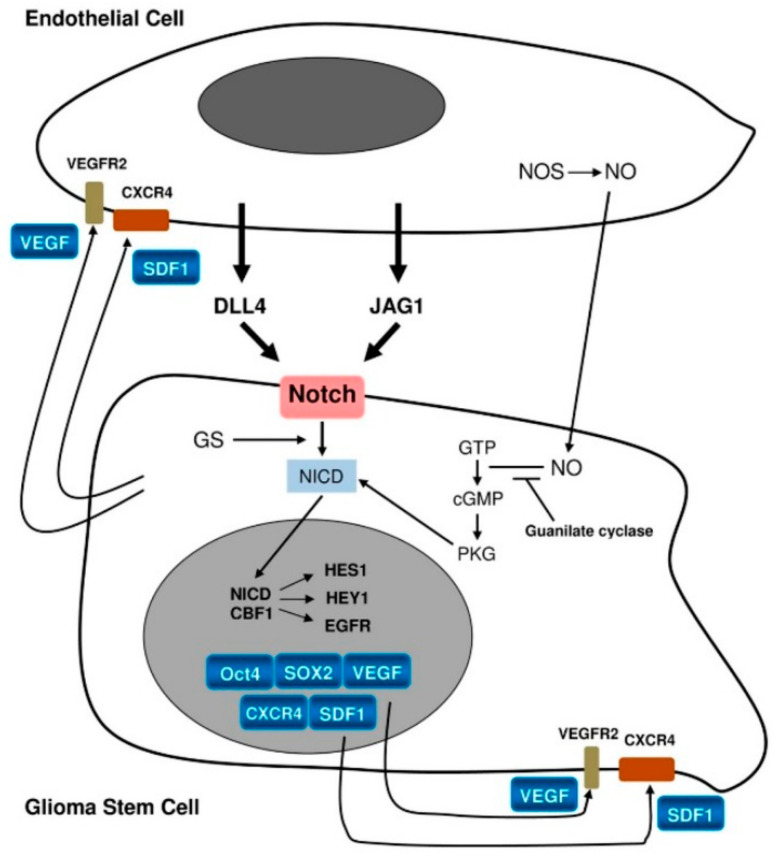
Schematic representation of interactions between the endothelium and glioma stem cells [26]. License of this figure provided at https://creativecommons.org/licenses/by/4.0/ (accessed on 19 February 2021). Changes made: figure description.

While ligands for the Notch pathway, e.g., Delta-like ligand 4 (DLL4) and Jagged1 (JAG1), are expressed on the endothelium, Notch-1 and Notch-2 themselves are expressed on GSCs [25]. Notch activation by these ligands (Figure 2) leads to activation of the target genes *Hes1* and *Hey1*. This represents one of the mechanisms promoting GSCs [25]. It has been confirmed by Notch pathway blockade, which was found to lead to reduced expression of stemness markers as well as inhibition of neurosphere formation in vitro [37,38]. In turn, GSCs promote endothelial proliferation through the production of vascular endothelial growth factor (VEGF), angiogenesis, homing of bone marrow-derived endothelial precursor cells, and transdifferentiation of GSCs into pericytes [17,25].

Hypoxia represents an essential feature of the microenvironment in glioblastoma. Hypoxia sustains the self-renewal of GSCs and even increases the pool of GSCs [25]. It also maintains proliferation, invasive growth, and the survival of malignant cells [39,40,41]. Resistance to treatment is enhanced by hypoxia via multiple mechanisms. Hypoxia inhibits free radicals, thus lessening the efficacy of radiation treatment [25]. Regarding chemotherapy, expression of the multi-drug resistance gene *MDR1*/*ABCB1* is upregulated by hypoxia.

Glioma-associated microglia/macrophages (GAMs) [25] mostly exhibit pro-tumor effects, as GSCs promote the M2 differentiation of GAMs through periostin secretion, which increases tumor growth via αvβ3 integrin [26,42,43]. Similarly, tumor-associated neutrophils and myeloid-derived suppressor cells promote the progression of GBM [27,28,29]. In contrast, dendritic cells present antigens, including tumor antigens, to T lymphocytes, inducing an antigen-specific immune response against the neoplasm [25]. Dendritic cells also regulate the balance between inflammatory and inhibitory immune reactions [31].

Pericytes support the blood vessels and are characterized by the expression of the platelet-derived growth factor receptor beta PDGFRβ, α-smooth muscle actin, neuron-glial antigen 2/chondroitin sulphate proteoglycan 4 (NG2/CSPG4), and desmin [26,32]. In glioblastoma, pericytes can develop from GSCs via mesenchymal differentiation in order to maintain tumor growth and the blood supply [44]. When angiogenesis develops in glial cancer, the pericyte count starts to grow in parallel with disruption of the blood–brain barrier. This feature can serve as a marker of neo-vascularization in the tumor [45].

The presence of reactive astrocytes within the tumor and at the invasive edge used to be a well-known nightmare in diagnostic pathology. Reactive gemistocytes in glioblastoma might suggest a secondary nature of the tumor, while astrocytes at the invasive front of low-grade glioma had to be distinguished from the neoplastic cells themselves in order to confirm the diagnosis of a glioma. These diagnostic difficulties can now be reliably solved via immunohistochemical detection of the mutant IDH1 protein IDH1-R132H. However, besides differential diagnostic considerations, the pathogenetic role of reactive astrocytes in GBM is under active study. Theoretically, two possibilities exist: reactive astrocytes might recognize glioma cells as having glial differentiation and support their proliferation; alternatively, astrocytes might try to counteract the invasive destruction of brain tissues. In scientific studies, multiple mechanisms have been recognized in terms of how reactive astrocytes facilitate the progression of a tumor. These processes include the production of matrix metalloproteinase 2, VEGF, and a wide spectrum of cytokines and growth factors (interleukin (IL) 6, insulin like growth factor 1 (IGF-1), IL-19). Modification of the microRNA landscape and glutamine metabolism as well as regulation of p53 and nuclear factor kappa-light-chain-enhancer of activated B cells (NF-κB) pathways benefit the development of glioma. The expression of gap junction channel protein 43 Cx43 contributes to chemoresistance. Invasion can be stimulated by cell volume changes due to modifications in H^+^ and Ca^2+^ flows [26,46]. Immunoprotection by astrocytes is an additional pro-tumor mechanism [25]. However, it has been noted by Schiffer that the supporting evidence for these glioma-facilitating mechanisms was obtained via in vitro experiments or under conditions when influence of the tumor itself cannot be excluded [25].

### 4.2. Host Immune System

Immunosuppression is a cardinal feature of malignant tumors, including brain neoplasms [17], where it might be further enhanced by the properties of the blood–brain barrier. GSCs contribute to the immunosuppressive environment of gliomas by inhibiting the activation of cytotoxic T lymphocytes, triggering apoptosis in them, and activating regulatory T lymphocytes. These processes are mediated through soluble galectin-3 and B7-H1 molecules [47]. Further, GSCs induce immunosuppressive M2 differentiation in GAMs via IL-10, transforming growth factor beta 1 (TGF-β1), soluble colony-stimulating factor 1 (sCSF-1), and macrophage inhibitory cytokine-1 (MIC-1) [48].

### 4.3. Metabolism

As previously discussed, GBM is characterized by a hypoxic environment. Besides the regulatory impact on the number and function of GSCs, hypoxia has profound metabolic effects, increasing the need for glycolysis, which is active in malignant cells, even under aerobic conditions [49].

Glioma cells, including GSCs, exhibit the Warburg effect [17]. This is characterized by a preference toward aerobic glycolysis rather than oxidative phosphorylation, which is more active in normal cells. Aerobic glycolysis is less effective in terms of ATP synthesis but beneficial as a metabolic source for the synthesis of new molecules [17].

Warburg’s observations were further elaborated in GSCs by Kathagen, who assessed the role of the pentose phosphate pathway (PPP). PPP activity is increased in actively proliferating normoxic cancer cells and decreased under a severe hypoxic state when invasion dominates [50]. Metabolism acts as a reciprocal switch between two pathways, glycolysis and PPP, and is associated with different behaviors, invasion versus proliferation, in accordance with the “go or grow” hypothesis [51]. Under hypoxic conditions, glycolysis is active, and cells exhibit active migration and invasion. Oxygenation activates the PPP and proliferation [48].

Hypoxia correlates with an insufficient supply of glucose. Under such conditions, GSCs are able to outcompete non-stem cells for glucose uptake through upregulation of the high-affinity neuronal glucose transporter GLUT [52]. GSCs have a reciprocal relationship with the nitric oxide synthase 2 (NOS2) enzyme, as they upregulate it and also depend on it to maintain their pathogenicity [53].

Metabolic processes are closely associated with the behavior of malignant cells, as was illustrated in the example of the “go or grow” hypothesis. However, the role of the metabolome is wider, including downstream and upstream associations with genetic and epigenetic processes. For instance, *IDH1* mutations are observed in a subset of gliomas. These genetic changes lead to a gain of enzymatic function, leading to accumulation of the oncometabolite 2-hydroxyglutarate which, in turn, inhibits demethylases TET1 and TET2, followed by DNA hypermethylation [17]. The extensive links between metabolic processes and tumor pathogenesis indicate that the metabolome is a promising target for innovative treatment.

### 4.4. Niche Factors

The niche concept was first used to describe the locations where normal neural stem cells are found. Later, analogous terminology was used to characterize the sites showing the highest density of GSCs in glioblastoma. In this regard, perivascular, invasive, and hypoxic niches are known. Perivascular niches develop along capillaries and arterioles where GSCs are in direct contact with the endothelium. Invasive niches are characterized by perivascular growth of single invasive neoplastic cells along the capillaries, between the endothelium and reactive astrocytes. The perivascular growth of nestin-positive GSCs results in the detachment of astrocyte end-feet from vessels and pericyte dissociation. These processes contribute to endothelial proliferation and angiogenesis, which is one of the hallmarks of GBM. Vascular occlusion and/or imbalance between the fast growth of GBM and slower proliferation of blood vessels leads to necrosis surrounded by densely cellular perinecrotic pseudopalisades. GSCs are present in these areas. They not only survive hypoxia but are also induced by hypoxia-inducible factors HIF-1 and HIF-2 as well as by hypoxia itself. Here, hypoxic perinecrotic niches develop [25]. The interaction between GSCs and the endothelium, vascular proliferation in glioblastoma, and necrosis represent different steps within the same process; therefore, it has been suggested that all niches are combined into the so-called hypoxic periarteriolar niche [54].

Different signaling pathways are active in the GSC niche, including the previously described Notch signaling [18,55,56]. Besides its interactions with the endothelium, the Notch cascade can become activated by the extracellular matrix protein tenascin. The triggering of Notch signaling is also facilitated by high numbers of activating receptors on GSCs, including inhibitor of differentiation 4 (ID4) and fatty-acid binding protein 7 (FABP7) [57]. Hypoxia-inducible factor 1α (HIF-1α) activates the Notch pathway via stabilization of its intracellular domain [54]. Indeed, active Notch signaling has been associated with increased invasion and migration capacities, as expected in a hypoxic environment in accordance with the “go or grow” hypothesis. NF-kB factor promotes resistance to radiation treatment in GBM [58]. The targeting of A20, which represents a regulating molecule in the NF-kB pathway, inhibits tumor growth and stem cell survival [59].

The Wnt pathway is involved in the stemness, angiogenesis, and invasiveness of tumor cells, while its physiological functions include embryological development of the CNS [60]. In GSCs, canonical and non-canonical activation of Wnt signaling develop via genetic and epigenetic mechanisms [55]. In glioblastoma, mutation of *FAT1* leads to abnormal activation of Wnt and promotes tumorigenesis [61]. CSC chromatin is dependent on the achaete-scute family basic helix-loop-helix transcription factor 1 (ASCL1), a transcription factor, that activates the Wnt pathway through the Dickkopf Wnt signaling inhibitor (DKK1) [62]. The Wnt cascade is also involved in resistance against temozolamide via the induction of MGMT (O6-methylguanine-DNA methyltransferase) expression, which preserves the genome from temozolomide-induced alkylation [55].

Finally, the Sonic hedgehog (Shh) pathway is activated in GSCs. The Shh cascade leads to activation of the glioma-associated oncogene *GLI1* and *GLI2* products, which bind to the Nanog promoter and upregulate this stem cell marker. The transcription factor activity of Nanog triggers the production of other stemness factors. In healthy tissues, Nanog is suppressed by p53, but in GBM, *TP53* function is frequently lost, further contributing to the activation of Shh and Nanog. Besides maintaining stem cell features, Shh is involved in drug resistance via upregulation of the drug efflux P-glycoprotein and other ATP-binding cassette transporters [55].

### 4.5. Genetic and Epigenetic Factors

Genes most commonly mutated in glioblastoma were first reported by The Cancer Genome Atlas (TCGA) Research Network in 2008 and then updated in 2014. In 2008, the published data revealed a comprehensive analysis of gene expression, DNA copy amount, and DNA methylation aberrations in 206 GBMs and nucleotide sequence aberrations from 91 cases of GBM. Eight genes (Table 1) were found to be mutated among the 91 examined glioblastomas [63]. In 2014, more than 500 GBMs were examined, and 71 mutated genes were identified. In addition to the first eight reported genes, leucine-zipper-like transcriptional regulator 1 (*LZTR1*) was identified as a novel significantly mutated gene. This gene has not previously been described in cancer but is associated with DiGeorge syndrome [63]. Inhibition of tumor sphere formation was observed as a result of the enforced expression of *LZTR1* in GSCs [64].

The epigenetic regulation of GSCs occurs via (1) DNA methylation; (2) alterations to the chromatin architecture that are either due to the post-translational modification of histones or the activity of polycomb group proteins; as well as (3) a modified spectrum of microRNA [17]. Abnormal DNA methylation may involve promoter areas (CpG islands), thus affecting gene expression. Promoter hypermethylation can silence tumor suppressor genes, e.g., *TP53*, while hypomethylation may activate oncogenes. The post-translational modification of histones involves methylation, acetylation, phosphorylation, and other reactions that result in epigenetic changes close to the promoter or enhancer areas of genes, again influencing the transcription of the involved genes. All of these mechanisms, including the upregulation of certain polycomb group proteins, e.g., BMI and EZH2, and the dysregulation of microRNAs, have been reported in GSCs [55].

Suva et al. reported on transcription factors that are able to transform non-stem glioblastoma cells into GSCs. Four essential transcription factors were described: SOX3, SALL2, OLIG2, and POU3F2 [81]. Among these, SALL2 and POU3F2 are newly discovered in the context of glioma. The transcription factor c-Myc is another key epigenetic regulator that activates a stem-like transcriptional module and is highly expressed in GSCs compared with non-stem glioma cells. Inhibition of c-Myc expression in GSCs induced apoptosis [82]. Epigenetic studies of GSCs can reveal new therapeutic targets. For instance, it was recently described that GSCs are sensitive to inhibition of the histone demethylases KDM4C and KDM7A, resulting in DNA damage and the death of GSCs but not non-stem glioma cells [83].

## 5. Molecular Markers of Glioma Stem Cells

Several markers are characteristic of GSCs (Table 2), including the cell surface markers CD133 (Prominin 1), CD15, CD44, integrin alpha α6, L1CAM, and A2B5 and the cytoplasmic proteins SOX2, Nanog, Olig2, Myc, Musashi1, and nestin [17]. Of note, many of these markers are expressed both in GSCs and neural stem cells. Among them, CD133 was the first but not universal marker.

## 6. Significance of GSCs in Treatment Resistance

Aggressive growth, early and almost inevitable recurrence, and poor prognosis are features of GBM that are driving the necessity for new investigations into therapy resistance and targets for treatment [91]. Studies of GSCs are revealing mechanisms of resistance and targets for intervention.

### 6.1. Chemotherapy Resistance Mechanisms in GSCs

Temozolomide-based chemotherapy is an accepted standard within the combined treatment of glioblastoma. However, recurrences are almost inevitable within the first year after treatment. This short period of remission suggests that a fraction of GBM cells survive the treatment. GSCs seem to be suitable candidates because of their quiescent status, implying low proliferation and thus low activity of DNA synthesis, factors that might be targeted by chemotherapy. Indeed, Liu et al. compared the impact of chemotherapy on CD133+ GSCs in comparison with CD133-negative GBM cells. GSCs featured more marked chemoresistance compared with CD133-negative tumor cells [92,93,94]. In addition to quiescence, GSCs benefit from a higher capacity to escape apoptosis and activate DNA repair in comparison with non-stem tumor cells [93,95].

**Figure 3:**
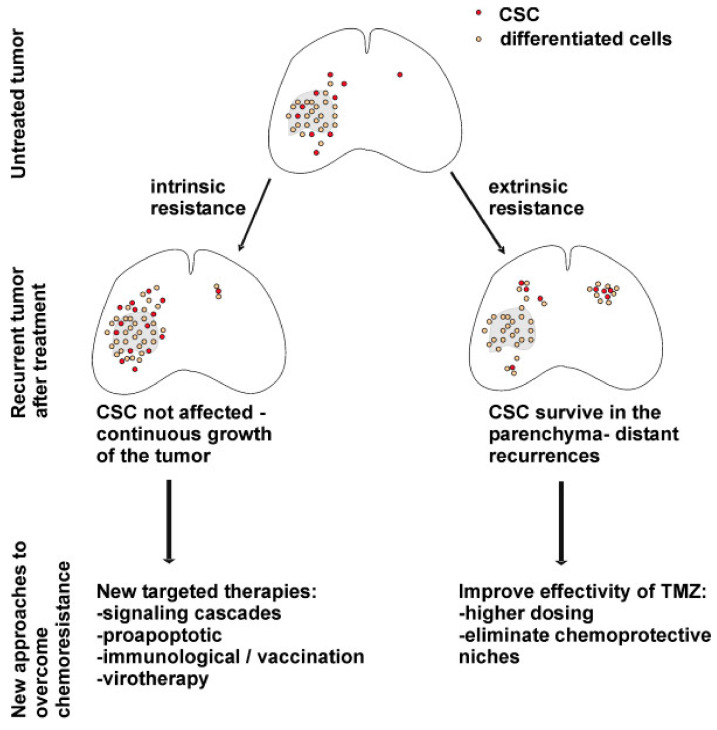
Behavior of GSCs, processes in the tumor in the context of intrinsic and extrinsic mechanisms of chemotherapy resistance and possible therapeutic strategies to overcome it [93]. License of this figure provided at https://creativecommons.org/licenses/by/2.0/ (accessed on 19 February 2021). Changes made: figure description.

Mechanisms of chemoresistance in glioblastoma are classified as intrinsic and extrinsic. Intrinsic factors refer to gene expression and molecular pathways within GSCs. Extrinsic factors include the properties of the tumor microenvironment, intercellular interactions, and the functionality of the blood–brain barrier [96]. The behaviors of GSCs in recurrent tumors and proposed therapeutic implications are depicted in Figure 3.

Activation of the Hedgehog (Hh) pathway and its downstream effector, glioma-associated oncogene 1 (GLI1), is associated with intrinsic resistance to temozolomide, which is partially mediated by insulin-like growth factor I (IGF-I). In turn, the suppression of GLI1 leads to restriction of IGF-I-dependent GSC proliferation, invasion, and angiogenesis [97].

O6-methylguanine-DNA methyltransferase (MGMT) is one of the most important enzymes responsible for promoting chemotherapy resistance in GSCs [96]. Under temozolomide treatment, significantly higher doses are necessary to accomplish therapeutic effect in MGMT-positive GSCs [98]. Temozolomide concentrations reached in patients were sufficient to eliminate GSCs in MGMT-negative tumors and, on the contrary, insufficient in MGMT-positive ones [98].

**Figure 4:**
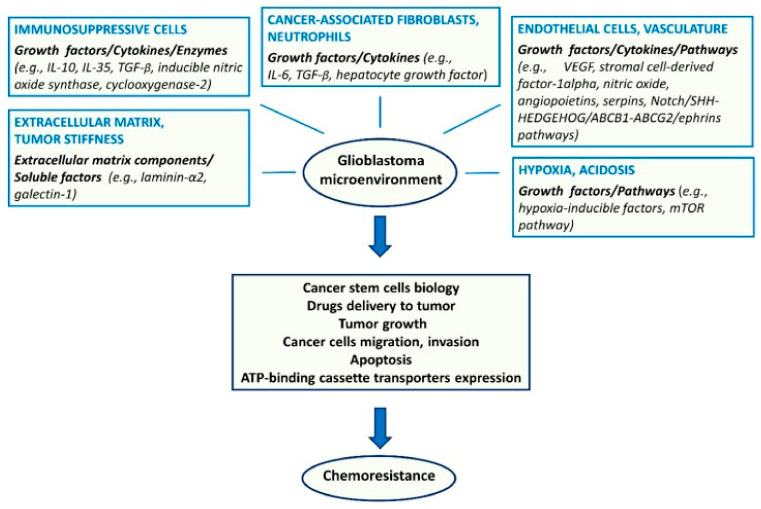
Major components of the tumor microenvironment and key molecular factors and pathways affecting glioma stem cell biology and chemoresistance [96]. License of this figure provided at https://creativecommons.org/licenses/by/4.0/ (accessed on 19 February 2021). Changes made: figure description.

Regarding extrinsic resistance mechanisms, the endothelium, other TME components (Figure 4), and hypoxia are of importance. Endothelial cells secrete angiopoietins, NO, and stromal cell derived factor 1α (SDF1α), which sustain GSCs and their resistance to therapy [99]. Besides the metabolic effects, hypoxia contributes to resistance through the activation of adenosine receptors [100].

Targeting GSCs has been found to be an effective measure to overcome temozolomide resistance and enhance its efficacy. The use of monoclonal antibody 8B6 was proposed to eradicate GSC-driven temozolomide resistance by targeting O-acetyl GD2 ganglioside, which is extensively expressed in glioma CSCs [101]. Immunotherapy with 8B6 was found to enhance temozolomide efficacy, decrease the expression of the GSC markers CD133 and nestin, and intensify tumor cell death in vitro and in vivo. The study is a proof of concept for combination therapy comprising chemo- and immunotherapy. In addition, this is pathogenetic evidence of the key role of GSCs in the treatment resistance of GBM [98].

Alternatively, GSCs can be targeted by kinase inhibitors, e.g., by glycogen synthase kinase (GSK3β) inhibitor kenpaullone, which was selected from 1301 drugs that were evaluated for their effects on CSCs in glioblastoma [102]. In vitro, combined treatment was shown to decrease stemness and cell proliferation. In mouse models, kenpaullone, in combination with temozolomide, markedly increased survival when compared with treatment with temozolomide alone [99].

### 6.2. Radiotherapy Resistance Mechanisms in Glioma CSCs

Similar to temozolomide, therapeutic irradiation is an essential component of the glioblastoma treatment protocol, but it cannot prevent recurrences. CD133-positive GSCs exhibit markedly increased resistance compared with CD133-negative glioma cells, both in vitro and in vivo. CD133-expressing cells are able to activate the DNA damage checkpoint and rapidly repair the damage [21].

A study by Carruthers et al. underlined the concept of replication stress (RS) as one of the key mechanisms in DNA damage control by glioma stem cells. RS is defined as inefficient DNA replication that causes replication forks to progress slowly or to stop. RS activates certain molecular events to stabilize the replication forks and prevent DNA damage that might be caused by RS. Artificially induced RS in glioma cells is associated with radio resistance. GSCs exhibit constitutively increased RS, as is evident from elevated levels of the following RS markers: replication protein A, single-stranded DNA binding protein, and DNA damage markers [16,100]. RS in GSCs might be attributable to the transcription of long neural genes. Very large genes can be transcribed over more than one cell cycle, thus extending their transcription into the S phase which, in turn, leads to possible collision between replication and transcription and the formation of DNA–RNA hybrids or the so-called R-loop. These hybrids develop if transcribed RNA molecules undergo hybridization with their complementary DNA strands, displacing the other DNA molecule as a single-stranded DNA. Thus, GSCs express long neural genes and produce RNA–DNA hybrids, supporting the assumption that altered transcription/replication results in RS in glioma stem cells. Ataxia telangiectasia and Rad3-related kinase (ATR) and checkpoint kinase 1 (CHK1), responsible for DNA break repair, are activated in GSCs. The blockage of ATR and poly-(ADP-ribose)-polymerase PARP has been shown to be effective for the eradication of radio resistance. Hence, these enzymes are notable both as pathogenetically important mechanisms of resistance against irradiation and treatment targets to ensure radio sensitization [17,103].

Regarding the signaling cascade of DNA damage repair under ionizing radiation, tyrosine kinase MET should also be mentioned. It is essential for normal cell migration during embryonic development [104]. In cancer, it contributes to cell survival, angiogenesis, invasion, and metastasis [105]. MET induces radio resistance through the activation of AKT kinase and the subsequent downstream effectors of DNA repair. The other MET-induced mechanism of radio resistance includes phosphorylation and cytoplasmic retention of p21 protein, which has an anti-apoptotic effect. Inhibition of MET can induce radio sensitivity in GSCs [106].

The significance of radio resistant GSCs is not limited to a few surviving cells that later give rise to recurrence. Both clinical outcomes and mathematical modeling show that GSCs are a key mechanism in determining the resistance of the whole tumor to therapy. Isolated cell lines of glioblastoma do not show so marked resistance, as observed in GBM patients, and this suggests that cellular interplay is mandatory for biological resistance. In mathematical modelling, treatment failure by conventional and hypofractionated radiation treatment can be explained only by an ordinary differential equation that accounts for the dynamic interaction between GSCs and non-stem glioma cells, which exhibit different levels of sensitivity to radiation-induced damage [107].

## 7. Insights into Innovative Glioma Treatment Approaches Targeting GSCs

Current treatments of gliomas target actively proliferating cells and the bulk tumor mass [104]. GSC-oriented approaches are a promising way to overcome resistance and lower the risk of recurrence by eliminating the quiescent cells surviving the first line of treatment and the cellular crosstalk between GSCs and non-stem cells [55].

### 7.1. Pharmacological Targeting of Molecular Pathways Inducing GSC Tumorigenicity and Chemoresistance

Wnt/β-catenin signaling leads to the activation of target genes, which are responsible for the maintenance of stemness. The core event in this cascade is the stabilization of β-catenin, allowing it to enter the nucleus and bind to T cell factor [55]. This signaling pathway can be inhibited by the cyclooxygenase-2 inhibitor celecoxib and the small molecules XAV939 and SEN461, which stabilize axin. Axin contributes to β-catenin destruction and increases the amount of phosphorylated β-catenin in the cytoplasm so that it is not able to enter the nucleolus [55].

Hedgehog signaling is controlled by the transmembrane receptors Patched1 (PTCH1) and Smoothened (SMO), which exhibit inhibitory and activating functions, respectively. In the absence of an activating ligand, PTCH1 keeps SMO inactive. When the Hh pathway becomes activated by the attachment of sonic Hedgehog Shh to the corresponding receptor, PTCH1, SMO is released, resulting in activation of the downstream effector glioma-associated oncogene 1 GLI1 [8,108]. GLI1 enters the nucleolus and induces the transcription of genes responsible for cellular self-renewal, proliferation, and chemoresistance. The Hh pathway can be blocked by arsenic trioxide (which is used for the treatment of acute promyelocytic leukemia and solid tumors) as well as by vismodegib, representing a small molecule inhibitor of SMO. An isoflavone, representing a combined pharmacophore capable of targeting both SMO and GLI1, has also been described [8,107,108].

As previously mentioned, the Notch pathway is important in GSCs. Hypothetically, it can be blocked by tarextumab, a monoclonal antibody that binds to Notch receptors, thus at least partially blocking the relevant signaling pathway [55]. Alternatively, Notch blockade can be achieved via inhibition of gamma secretase activity that is necessary to split off the intracellular domain of Notch (after the receptor has been engaged with the ligand) and to trigger the further cascade of intranuclear events, including CDF1-activated transcription of *Hes* and *Hey* genes [37,38].

### 7.2. Immunological and Metabolic Intervention Targeting GSCs

#### 7.2.1. Immunotherapy for Glioblastoma

Regarding the immunological treatment of GBM, different options are being investigated, including the use of cytotoxic T lymphocytes, tumor vaccination, and immune modulation with antibodies against PD1, PD-L1, and CTLA4 [17]. Natural killer (NK) lymphocytes represent a particularly promising field of study. NK cells are highly cytotoxic and more capable of withstanding elimination from the tumor microenvironment, compared with other immune or inflammatory cells. Autologous NK cells as well as IL-2 activated NKs have been tested in glioma patient trials. The use of allogenic NKs or antibodies against KIR receptors would be helpful to prevent the recognition of self-MHC class I molecules on glioblastoma cells. NKs can also be combined with antibodies targeting angiogenesis or epidermal growth factor receptor on glioma cells [109]. Regarding GSCs, NKs that carry the chimeric antigen receptor (CAR) and target HER2 are capable of eliminating glioma cells and neurospheres [106].

NK group 2 member D (NKG2D) ligands are overexpressed in GBM and can be targeted by CAR-T cells [110]. The effects of these T lymphocytes were tested on glioma tumor cells and stem cells in vitro and in vivo using xenografts. CAR-T cells produced perforin, granzyme B, and high amounts of cytokines in vitro and successfully induced the lysis of GSCs and bulk tumor cells. In vivo, T cells markedly abolished xenograft tumors and lacked significant toxicity [107]. Human leukocyte antigen (HLA)-presented peptidomes in GSCs have been assessed as well. Numerous HLA ligands have been distinguished, and these may serve as targets for CD8+ T cell immunotherapy [111]. Stemness markers also can be targeted by immune means, e.g., by cytotoxic avian IgY-immunotoxin against human CD133+ GSCs. In vitro, the IgY toxin was found to diminish cell viability by 55%, and in vivo it was associated with a decrease in tumor volume of more than 50% [112].

#### 7.2.2. Targeting Metabolism in Glioma and GSCs

Gliomas and GSCs exhibit a spectrum of metabolic changes that are closely and bidirectionally linked with genetic events. GSCs express the high-affinity glucose transporter GLUT3, giving them a survival benefit in hypoglycemic microenvironments. Downstream of GLUT3, the influx of carbon is directed into de novo purine synthesis. De novo synthesis of pyrimidine is also upregulated in GSCs in comparison with non-stem glioma cells. Rate-limiting enzymes that are involved in pyrimidine synthesis are essential to maintain stemness. Targeting pyrimidine synthesis in GSCs inhibits stem cell survival, tumor initiation, and self-renewal in vivo [113].

Mutated isocitrate dehydrogenase 1 (IDH1) is another possible treatment target [9]. Unfortunately, *IDH* is mutated in secondary GBM; therefore, IDH-blocking treatment would not be universal [114]. IDH is an essential enzyme in a wide range of physiologic processes, such as metabolism, epigenetic regulation, and DNA repair [115]. In vitro, the overexpression of IDH1^R132H^ and IDH2^R172K^ mutant proteins in glioblastoma cells led to increased radiosensitivity and suppressed tumor cell growth and migration [116]. This is in accordance with higher efficacy of radiotherapy in proneural glioblastoma, comprising *IDH1* mutant cases, in comparison with the mesenchymal subtype of GBM, which is enriched in CD44 [11].

#### 7.2.3. Tumoricidal Neural Stem Cell Therapy

Completely new strategies for GBM treatment are on the way, e.g., tumoricidal neural stem cell therapy. These types of treatment were designed to overcome intratumoral heterogeneity regarding invasiveness and response to medications. At first, authors generated novel GBM models using organotypic brain slice explants and distinct human GBM cell types. After that, the efficacy of primary neural stem cells and fibroblast-derived human induced neural stem cells engineered with tumor necrosis factor-related apoptosis-inducing ligand (TRAIL) was tested and found to inhibit tumor growth and increase survival [117].

## 8. Conclusions

Glioblastoma is the most frequent malignant brain tumor. It is characterized by a dismal prognosis and a low, but growing, incidence. The gold standard of glioblastoma treatment includes surgical resection, followed by radiotherapy combined with concomitant or adjuvant chemotherapy with temozolomide. However, the best treatment results in a median survival time of 14–21 months. Recurrences are early and almost inevitable. Glioma stem cells, characterized by the expression of CD133 and nestin, represent a mainstay in the pathogenesis of treatment resistance and recurrences. Regarding the origin of GSCs, two hypotheses have been proposed. Malignant changes in neural stem cells have been shown experimentally and might be promoted by replication stress. Dedifferentiation of non-stem glioblastoma cells, reaching dynamic equilibrium with GSCs, can also develop. Currently, two molecular GSC subtypes are known: mesenchymal and proneural. GSCs are subjected to complex regulation by genetic, epigenetic, and metabolic alterations, the immune system, the tumor microenvironment, and hypoxic periarteriolar niche factors. The chemoresistance of GSCs is promoted by quiescence, active DNA repair, and a greater ability to escape apoptosis. Wnt/β-catenin, Hedgehog and Notch pathways, upregulation of MGMT, and ATP-binding cassette transporter proteins are important molecular mechanisms. Radio resistance is based on constitutively elevated replication stress and the MET cascade. Mathematical modeling points toward the crucial importance of GSC eradication to overcome the treatment failures in glioblastoma by interrupting cellular interactions in the tumor microenvironment. Molecular, immunological, and metabolic approaches to the targeting of GSCs are under development.

## Figures and Tables

**Table 1:** Most commonly mutated genes in glioblastoma: general functions and roles in glioma stem cells.

Gene	Function	Role in GSCs	Ref.
***TP53***	Critical tumor suppressor gene, induces apoptosis.	Promotes migration and self-renewal of GSCs.	[65,66]
***PTEN***	Tumor suppressor gene, regulates proliferation and apoptosis.	Induces malignant phenotype in neural stem cells.	[67,68]
***NF1***	Encodes neurofibromin, which inhibits Ras proliferative signaling. Tumor suppressor gene.	Promotes malignant change to glioma in oligodendrocyte progenitor cells.	[69,70]
***EGFR***	Regulates homeostasis and epithelial tissue genesis. Essential growth factor in embryogenesis.	Maintains GSCs by the AKT (Protein kinase B) pathway.	[18,71]
***IDH1***	Produces NADPH.	Expression correlates with aggressive phenotype in GSCs.	[72,73]
***RB1***	Encodes tumor suppressor protein, which regulates the cell cycle.	In the PDGFRα/Stat3/Rb1 signaling pathway, the depletion of PDGFRα expression in GSCs induces RB1 action. This finding has therapeutic value.	[74,75]
***PIK3R1***	Promotes migration and proliferation in cells as well as survival.	Altered expression of PIK3R1 induces malignant transformation of normal astrocytes in vivo.	[76,77]
***PIK3CA***	Promotes cell migration, proliferation, and survival.	Differentially promotes the development of glioma, based on the mutated domain. Mutated form is capable of inducing stemness.	[78,79,80]

*TP53*—tumor protein 53 gene, *PTEN*—phosphatase and tensin homolog gene, *NF1*—neurofibromin 1, *EGFR*—epidermal growth factor receptor gene, *IDH1*—isocitrate dehydrogenase 1, *RB1*—retiblastoma transcriptional corepressor 1, *PIK3R1*—phosphoinositide-3-kinase regulatory subunit 1, *PIK3CA*—phosphatidylinositol-4,5-bisphosphate 3-kinase catalytic subunit alpha.

**Table 2:** Markers of glioma stem cells.

GSC Marker	Significance	Ref.
**CD133** **(Prominin-1)**	First marker used to identify cancer stem cells (CSCs) in human brain tumors. CD133+ cells were able to produce tumors in immunocompromised mice and form tumor spheres in vitro.	[84,85]
**Nestin**	Nestin + cells show an increased capacity to form tumor spheres.	[86]
**SSEA-1**	CD133+ human GSCs show SSEA-1 co-expression.	[87,88]
**Integrin-α6**	Expressed in GSCs at high levels. Blockage of Integrin-α6 inhibits the tumor-initiating capacity and self-renewal.	[89]
**A2B5**	Strongly associated with tumor initiation in vivo.	[90]

## Data Availability

Not applicable.

## References

[1] Sung H., Ferlay J., Siegel R.L., Laversanne M., Soerjomataram I., Jemal A., Bray F. (2021). Global cancer statistics 2020: GLOBOCAN estimates of incidence and mortality worldwide for 36 cancers in 185 countries. CA Cancer J. Clin..

[2] Forjaz G., Sloan B.J.S., Kruchko C., Siegel R., Negoita S., Ostrom Q.T., Dickie L., Ruhl J., Van Dyke A., Patil N. (2021). An updated histology recode for the analysis of primary malignant and nonmalignant brain and other central nervous system tumors in the Surveillance, Epidemiology, and End Results Program. Neuro-Oncol. Adv..

[3] Liu S., Zhang C., Wang B., Zhang H., Qin G., Li C., Cao L., Gao Q., Ping Y., Zhang K. (2021). Regulatory T cells promote glioma cell stemness through TGF-β–NF-κB–IL6–STAT3 signaling. Cancer Immunol. Immunother..

[4] Ho V.K.Y., Reijneveld J.C., Enting R.H., Bienfait H.P., Robe P., Baumert B.G., Visser O. (2014). Changing incidence and improved survival of gliomas. Eur. J. Cancer.

[5] Philips A., Henshaw D.L., Lamburn G., Carroll O.M.J. (2018). Brain Tumours: Rise in Glioblastoma Multiforme Incidence in England 1995–2015 Suggests an Adverse Environmental or Lifestyle Factor. J. Environ. Public Health.

[6] Hardell L., Carlberg M., Mild K.H. (2005). Use of cellular telephones and brain tumour risk in urban and rural areas. Occup. Environ. Med..

[7] Stupp R., Hegi M.E., Mason W.P., Bent M.J.V.D., Taphoorn M.J.B., Janzer R.C., Ludwin S.K., Allgeier A., Fisher B., Belanger K. (2009). Effects of radiotherapy with concomitant and adjuvant temozolomide versus radiotherapy alone on survival in glioblastoma in a randomised phase III study: 5-year analysis of the EORTC-NCIC trial. Lancet Oncol..

[8] Bureta C., Saitoh Y., Tokumoto H., Sasaki H., Maeda S., Nagano S., Komiya S., Taniguchi N., Setoguchi T. (2019). Synergistic effect of arsenic trioxide, vismodegib and temozolomide on glioblastoma. Oncol. Rep..

[9] Garnier D., Renoult O., Alves-Guerra M.-C., Paris F., Pecqueur C. (2019). Glioblastoma Stem-Like Cells, Metabolic Strategy to Kill a Challenging Target. Front. Oncol..

[10] Bozzato E., Bastiancich C., Préat V. (2020). Nanomedicine: A Useful Tool against Glioma Stem Cells. Cancers.

[11] Jakovlevs A., Vanags A., Gardovskis J., Strumfa I. (2019). Molecular classification of diffuse gliomas. Pol. J. Pathol..

[12] Stoyanov G.S., Dzhenkov D.L. (2018). On the Concepts and History of Glioblastoma Multiforme–Morphology, Genetics and Epigenetics. Folia Medica.

[13] Lee J.S., Lee H.J., Moon B.H., Song S.H., Lee M.O., Shim S.H., Kim H.S., Lee M.C., Kwon J.T., Fornace A.J. (2012). Generation of Cancerous Neural Stem Cells Forming Glial Tumor by Oncogenic Stimulation. Stem Cell Rev. Rep..

[14] Morvinski F.D., Bushong E.A., Ke E., Soda Y., Marumoto T., Singer O., Ellisman M.H., Verma I.M. (2012). Dedifferentiation of Neurons and Astrocytes by Oncogenes Can Induce Gliomas in Mice. Science.

[15] Sassi F.D.A., Brunetto A.L., Schwartsmann G., Roesler R., Abujamra A.L. (2012). Glioma Revisited: From Neurogenesis and Cancer Stem Cells to the Epigenetic Regulation of the Niche. J. Oncol..

[16] Siebzehnrubl F.A., Reynolds B.A., Vescovi A., Steindler D.A., Deleyrolle L.P. (2011). The origins of glioma: E Pluribus Unum?. Glia.

[17] Carruthers R.D., Ahmed S.U., Ramachandran S., Strathdee K., Kurian K.M., Hedley A., Roman G.N., Kalna G., Neilson M.P., Gilmour L. (2018). Replication Stress Drives Constitutive Activation of the DNA Damage Response and Radioresistance in Glioblastoma Stem-like Cells. Cancer Res..

[18] Lathia J.D., Mack S.C., Hubert M.E., Valentim C.L., Rich J.N. (2015). Cancer stem cells in glioblastoma. Genes Dev..

[19] Eramo A., Vitiani L.R., Zeuner A., Pallini R., Lotti F., Sette G., Pilozzi E., La Rocca L.M., Peschle C., De Maria R. (2006). Chemotherapy resistance of glioblastoma stem cells. Cell Death Differ..

[20] Chen J., Li Y., Yu T.S., McKay R.M., Burns D.K., Kernie S.G., Parada L.F. (2012). A restricted cell population propagates glioblastoma growth after chemotherapy. Nat. Cell Biol..

[21] Bao S., Wu Q., McLendon R.E., Hao Y., Shi Q., Hjelmeland A.B., Dewhirst M.W., Bigner D.D., Rich J.N. (2006). Glioma stem cells promote radioresistance by preferential activation of the DNA damage response. Nature.

[22] Verhaak R.G., Hoadley K.A., Purdom E., Wang V., Qi Y., Wilkerson M.D., Miller C.R., Ding L., Golub T., Mesirov J.P. (2010). Integrated Genomic Analysis Identifies Clinically Relevant Subtypes of Glioblastoma Characterized by Abnormalities in PDGFRA, IDH1, EGFR, and NF. Cancer Cell.

[23] Popova S.N., Bergqvist M., Dimberg A., Edqvist P., Ekman S., Hesselager G., Ponten F., Smits A., Sooman L., Alafuzoff I. (2013). Subtyping of gliomas of various WHO grades by the application of immunohistochemistry. Histopathology.

[24] Āboliņš A., Vanags A., Trofimovičs G., Miklaševičs E., Gardovskis J., Štrumfa I. (2011). Molecular subtype shift in breast cancer upon trastuzumab treatment: A case report. Pol. J. Pathol..

[25] Guardia G.D.A., Correa B.R., Araujo P.R., Qiao M., Burns S., Penalva L.O.F., Galante P.A.F. (2020). Proneural and mesenchymal glioma stem cells display major differences in splicing and lncRNA profiles. NPJ Genom. Med..

[26] Schiffer D., Annovazzi L., Casalone C., Corona C., Mellai M. (2018). Glioblastoma: Microenvironment and Niche Concept. Cancers.

[27] Liang J., Piao Y., Holmes L., Fuller G.N., Henry V., Tiao N., De Groot J.F. (2014). Neutrophils Promote the Malignant Glioma Phenotype through S100A. Clin. Cancer Res..

[28] Kohanbash G., Okada H. (2012). Myeloid-derived Suppressor Cells (MDSCs) in Gliomas and Glioma-Development. Immunol. Investig..

[29] Feng X., Szulzewsky F., Yerevanian A., Chen Z., Heinzmann D., Rasmussen R.D., Garcia A.V., Kim Y., Wang B., Tamagno I. (2015). Loss of CX3CR1 increases accumulation of inflammatory monocytes and promotes gliomagenesis. Oncotarget.

[30] Ho I.A.W., Shim W.S.N. (2017). Contribution of the Microenvironmental Niche to Glioblastoma Heterogeneity. BioMed Res. Int..

[31] Mineharu Y., Castro M.G., Lowenstein P.R., Sakai N., Miyamoto S. (2013). Dendritic Cell-Based Immunotherapy for Glioma: Multiple Regimens and Implications in Clinical Trials. Neurol. Med. Chir..

[32] Raza A., Franklin M.J., Dudek A.Z. (2010). Pericytes and vessel maturation during tumor angiogenesis and metastasis. Am. J. Hematol..

[33] Hira V.V., Wormer J.R., Kakar H., Breznik B., Van Der Swaan B., Hulsbos R., Tigchelaar W., Tonar Z., Khurshed M., Molenaar R.J. (2018). Periarteriolar Glioblastoma Stem Cell Niches Express Bone Marrow Hematopoietic Stem Cell Niche Proteins. J. Histochem. Cytochem..

[34] Charles N.A., Holland E.C., Gilbertson R., Glass R., Kettenmann H. (2011). The brain tumor microenvironment. Glia.

[35] Schiffer D., Annovazzi L., Mazzucco M., Mellai M. (2015). The Microenvironment in Gliomas: Phenotypic Expressions. Cancers.

[36] Hambardzumyan D., Becher O.J., Rosenblum M.K., Pandolfi P.P., Todorova M.K., Holland E.C. (2008). PI3K pathway regulates survival of cancer stem cells residing in the perivascular niche following radiation in medulloblastoma in vivo. Genes Dev..

[37] Fan X., Khaki L., Zhu T.S., Soules M.E., Talsma C.E., Gul N., Koh C., Zhang J., Li Y.M., Maciaczyk J. (2009). Notch Pathway Blockade Depletes CD133-Positive Glioblastoma Cells and Inhibits Growth of Tumor Neurospheres and Xenografts. Stem Cells.

[38] Xu R., Shimizu F., Hovinga K., Beal K., Karimi S., Droms L., Peck K.K., Gutin P., Iorgulescu J.B., Kaley T. (2016). Molecular and Clinical Effects of Notch Inhibition in Glioma Patients: A Phase 0/I Trial. Clin. Cancer Res..

[39] Hanahan D., Weinberg R.A. (2011). Hallmarks of Cancer: The Next Generation. Cell.

[40] Fischer U., Radermacher J., Mayer J., Mehraein Y., Meese E. (1992). Tumor hypoxia: Impact on gene amplification in glioblastoma. Int. J. Oncol..

[41] Binello E., Germano I.M. (2011). Targeting glioma stem cells: A novel framework for brain tumors. Cancer Sci..

[42] Kennedy B.C., Showers C.R., Anderson D.E., Anderson L., Canoll P., Bruce J.N., Anderson R.C.E. (2013). Tumor-Associated Macrophages in Glioma: Friend or Foe?. J. Oncol..

[43] Zhou W., Ke S.Q., Huang Z., A Flavahan W., Fang X., Paul J., Wu L., Sloan A.E., McLendon R.E., Li X. (2015). Periostin secreted by glioblastoma stem cells recruits M2 tumour-associated macrophages and promotes malignant growth. Nat. Cell Biol..

[44] Cheng L., Huang Z., Zhou W., Wu Q., Donnola S., Liu J.K., Fang X., Sloan A.E., Mao Y., Lathia J.D. (2013). Glioblastoma Stem Cells Generate Vascular Pericytes to Support Vessel Function and Tumor Growth. Cell.

[45] Schiffer D., Mellai M., Bovio E., Bisogno I., Casalone C., Annovazzi L. (2018). Glioblastoma niches: From the concept to the phenotypical reality. Neurol. Sci..

[46] Guan X., Hasan N., Maniar S., Jia W., Sun D. (2018). Reactive Astrocytes in Glioblastoma Multiforme. Mol. Neurobiol..

[47] Wei J., Barr J., Kong L.Y., Wang Y., Wu A., Sharma A.K., Gumin J., Henry V., Colman H., Sawaya R. (2010). Glioma-Associated Cancer-Initiating Cells Induce Immunosuppression. Clin. Cancer Res..

[48] Wu A., Wei J., Kong L.Y., Wang Y., Priebe W., Qiao W., Sawaya R., Heimberger A.B. (2010). Glioma cancer stem cells induce immunosuppressive macrophages/microglia. Neuro-Oncology.

[49] Li Z., Bao S., Wu Q., Wang H., Eyler C., Sathornsumetee S., Shi Q., Cao Y., Lathia J., McLendon R.E. (2009). Hypoxia-Inducible Factors Regulate Tumorigenic Capacity of Glioma Stem Cells. Cancer Cell.

[50] Buhmann K.A., Schulte A., Weller J., Holz M., Mende H.C., Glass R., Lamszus K. (2016). Glycolysis and the pentose phosphate pathway are differentially associated with the dichotomous regulation of glioblastoma cell migration versus proliferation. Neuro-Oncology.

[51] Kathagen A., Schulte A., Balcke G., Phillips H.S., Martens T., Matschke J., Günther H.S., Soriano R., Modrusan Z., Sandmann T. (2013). Hypoxia and oxygenation induce a metabolic switch between pentose phosphate pathway and glycolysis in glioma stem-like cells. Acta Neuropathol..

[52] Flavahan W.A., Wu Q., Hitomi M., Rahim N., Kim Y., Sloan A.E., Weil R.J., Nakano I., Sarkaria J.N., Stringer B.W. (2013). Brain tumor initiating cells adapt to restricted nutrition through preferential glucose uptake. Nat. Neurosci..

[53] Eyler C.E., Wu Q., Yan K., MacSwords J.M., Militello C.D., Misuraca K.L., Lathia J.D., Forrester M.T., Lee J., Stamler J.S. (2011). Glioma Stem Cell Proliferation and Tumor Growth Are Promoted by Nitric Oxide Synthase. Cell.

[54] Breznik B., Stokin C.L., Kos J., Khurshed M., Hira V.V.V., Bošnjak R., Lah T.T., Van Noorden C.J.F. (2018). Cysteine cathepsins B, X and K expression in peri-arteriolar glioblastoma stem cell niches. J. Mol. Histol..

[55] Kopan R. (2012). Notch Signaling. Cold Spring Harb. Perspect. Biol..

[56] Qiang L., Wu T., Zhang H.W., Lu N., Hu R., Wang Y.J., Zhao L., Chen F.H., Wang X.T., You Q.D. (2011). HIF-1α is critical for hypoxia-mediated maintenance of glioblastoma stem cells by activating Notch signaling pathway. Cell Death Differ..

[57] Sharifzad F., Ghavami S., Verdi J., Mardpour S., Sisakht M.M., Azizi Z., Taghikhani A., Łos M.J., Fakharian E., Ebrahimi M. (2019). Glioblastoma cancer stem cell biology: Potential theranostic targets. Drug Resist. Updat..

[58] Bhat K.P., Balasubramaniyan V., Vaillant B., Ezhilarasan R., Hummelink K., Hollingsworth F., Wani K., Heathcock L., James J.D., Goodman L.D. (2013). Mesenchymal Differentiation Mediated by NF-κB Promotes Radiation Resistance in Glioblastoma. Cancer Cell.

[59] Hjelmeland A.B., Wu Q., Wickman S., Eyler C., Heddleston J., Shi Q., Lathia J.D., MacSwords J., Lee J., McLendon R.E. (2010). Targeting A20 Decreases Glioma Stem Cell Survival and Tumor Growth. PLoS Biol..

[60] Mccord M., Mukouyama Y.S., Gilbert M.R., Jackson S. (2017). Targeting WNT Signaling for Multifaceted Glioblastoma Therapy. Front. Cell. Neurosci..

[61] Morris L.G., Kaufman A.M., Gong Y., Ramaswami D., Walsh L.A., Şevin T., Eng S., Kannan K., Zou Y., Peng L. (2013). Recurrent somatic mutation of FAT1 in multiple human cancers leads to aberrant Wnt activation. Nat. Genet..

[62] Rheinbay E., Suvà M.L., Gillespie S.M., Wakimoto H., Patel A.P., Shahid M., Oksuz O., Rabkin S.D., Martuza R.L., Rivera M.N. (2013). An Aberrant Transcription Factor Network Essential for Wnt Signaling and Stem Cell Maintenance in Glioblastoma. Cell Rep..

[63] Cancer Genome Atlas Research Network (2008). Comprehensive genomic characterization defines human glioblastoma genes and core pathways. Nat. Cell Biol..

[64] Brennan C.W., Verhaak R.G.W., McKenna A., Campos B., Noushmehr H., Salama S.R., Zheng S., Chakravarty D., Sanborn J.Z., Berman S.H. (2013). The Somatic Genomic Landscape of Glioblastoma. Cell.

[65] Daniele S., Taliani S., Da Pozzo E., Giacomelli C., Costa B., Trincavelli M.L., Rossi L., La Pietra V., Barresi E., Carotenuto A. (2014). Apoptosis Therapy in Cancer: The First Single-molecule Co-activating p53 and the Translocator Protein in Glioblastoma. Sci. Rep..

[66] Zhu H., Wang H., Huang Q., Liu Q., Guo Y., Lu J., Li X., Xue C., Han Q. (2018). Transcriptional Repression of p53 by PAX3 Contributes to Gliomagenesis and Differentiation of Glioma Stem Cells. Front. Mol. Neurosci..

[67] Duan S., Yuan G., Liu X., Ren R., Li J., Zhang W., Wu J., Xu X., Fuchou T., Li Y. (2015). PTEN deficiency reprogrammes human neural stem cells towards a glioblastoma stem cell-like phenotype. Nat. Commun..

[68] Hopkins B.D., Hodakoski C., Barrows D., Mense S.M., Parsons R.E. (2014). PTEN function: The long and the short of it. Trends Biochem. Sci..

[69] Yap Y.S., McPherson J.R., Ong C.K., Rozen S.G., Teh B.T., Lee A.S.G., Callen D.F. (2014). The NF1 gene revisited—From bench to bedside. Oncotarget.

[70] Liu C., Sage J.C., Miller M.R., Verhaak R.G., Hippenmeyer S., Vogel H., Foreman O., Bronson R.T., Nishiyama A., Luo L. (2011). Mosaic Analysis with Double Markers Reveals Tumor Cell of Origin in Glioma. Cell.

[71] Sigismund S., Avanzato D., Lanzetti L. (2018). Emerging functions of the EGFR in cancer. Mol. Oncol..

[72] Liu A., Hou C., Chen H., Zong X., Zong P. (2016). Genetics and Epigenetics of Glioblastoma: Applications and Overall Incidence of IDH1 Mutation. Front. Oncol..

[73] Yao Q., Cai G., Yu Q., Shen J., Gu Z., Chen J., Shi W., Shi J. (2017). IDH1 mutation diminishes aggressive phenotype in glioma stem cells. Int. J. Oncol..

[74] Giacinti C., Giordano A. (2006). RB and cell cycle progression. Oncogene.

[75] Cenciarelli C., Marei H.E., Felsani A., Casalbore P., Sica G., Puglisi M.A., Cameron A.J., Olivi A., Mangiola A. (2016). PDGFRα depletion attenuates glioblastoma stem cells features by modulation of STAT3, RB1 and multiple oncogenic signals. Oncotarget.

[76] Genetics Home Reference (2019). “PIK3R1 Gene” Genetics Home Reference. https://www.medlineplus.gov/genetics/gene/pik3r1/.

[77] Quayle S.N., Lee J.Y., Cheung L.W.T., Ding L., Wiedemeyer R., Dewan R.W., Huang-Hobbs E., Zhuang L., Wilson R.K., Ligon K.L. (2012). Somatic Mutations of PIK3R1 Promote Gliomagenesis. PLoS ONE.

[78] Genetics Home Reference (2019). “PIK3CA Gene” Genetics Home Reference. https://www.medlineplus.gov/genetics/gene/pik3ca/.

[79] McNeill R.S., Stroobant E.E., Smithberger E., Canoutas D.A., Butler M.K., Shelton A.K., Patel S.D., Limas J.C., Skinner K.R., Bash R.E. (2018). PIK3CA missense mutations promote glioblastoma pathogenesis, but do not enhance targeted PI3K inhibition. PLoS ONE.

[80] Madsen R.R., Knox R.G., Pearce W., Lopez S., Mahler-Araujo B., McGranahan N., Vanhaesebroeck B., Semple R.K. (2019). OncogenicPIK3CApromotes cellular stemness in an allele dose-dependent manner. Proc. Natl. Acad. Sci. USA.

[81] Suvà M.L., Rheinbay E., Gillespie S.M., Patel A.P., Wakimoto H., Rabkin S.D., Riggi N., Chi A.S., Cahill D.P., Nahed B.V. (2014). Reconstructing and Reprogramming the Tumor-Propagating Potential of Glioblastoma Stem-like Cells. Cell.

[82] Wang J., Wang H., Li Z., Wu Q., Lathia J.D., McLendon R.E., Hjelmeland A.B., Rich J.N. (2008). c-Myc Is Required for Maintenance of Glioma Cancer Stem Cells. PLoS ONE.

[83] Mallm J., Windisch P., Biran A., Gal Z., Schumacher S., Glass R., Mende H.C., Meshorer E., Barbus M., Rippe K. (2019). Glioblastoma initiating cells are sensitive to histone demethylase inhibition due to epigenetic deregulation. Int. J. Cancer.

[84] Singh S.K., Clarke I.D., Terasaki M., E Bonn V., Hawkins C., Squire J., Dirks P.B. (2003). Identification of a cancer stem cell in human brain tumors. Cancer Res..

[85] Hemmati H.D., Nakano I., Lazareff J.A., Smith M.M., Geschwind D.H., Fraser B.M., Kornblum H.I. (2003). Cancerous stem cells can arise from pediatric brain tumors. Proc. Natl. Acad. Sci. USA.

[86] Jin X., Jin X., Jung J.E., Beck S., Kim H. (2013). Cell surface Nestin is a biomarker for glioma stem cells. Biochem. Biophys. Res. Commun..

[87] Son M.J., Woolard K., Nam D.-H., Lee J., Fine H.A. (2009). SSEA-1 Is an Enrichment Marker for Tumor-Initiating Cells in Human Glioblastoma. Cell Stem Cell.

[88] Ahmed A.U., Auffinger B., Lesniak M.S. (2013). Understanding glioma stem cells: Rationale, clinical relevance and therapeutic strategies. Expert Rev. Neurother..

[89] Lathia J.D., Gallagher J., Heddleston J.M., Wang J., Eyler C.E., MacSwords J., Wu Q., Vasanji A., McLendon R.E., Hjelmeland A.B. (2010). Integrin Alpha 6 Regulates Glioblastoma Stem Cells. Cell Stem Cell.

[90] Ogden A.T., Waziri A.E., Lochhead R.A., Fusco D., Lopez K., Ellis J.A., Kang J., Assanah M., McKhann G.M., Sisti M.B. (2008). Identification of A2b5+ Cd133− Tumor-Initiating Cells in Adult Human Gliomas. Neurosurgery.

[91] Kaynak O.E., Qutub A.A., Celiktas Y.O. (2018). Advances in Glioblastoma Multiforme Treatment: New Models for Nanoparticle Therapy. Front. Physiol..

[92] Liu G., Yuan X., Zeng Z., Tunici P., Ng H., Abdulkadir I.R., Lu L., Irvin D., Black K.L., Yu J.S. (2006). Analysis of gene expression and chemoresistance of CD133+ cancer stem cells in glioblastoma. Mol. Cancer.

[93] Ahmed S.I., Javed G., Laghari A.A., Bareeqa S.B., Farrukh S., Zahid S., Samar S.S., Aziz K. (2018). CD133 Expression in Glioblastoma Multiforme: A Literature Review. Cureus.

[94] Chen R., Nishimura M.C., Bumbaca S.M., Kharbanda S., Forrest W.F., Kasman I.M., Greve J.M., Soriano R.H., Gilmour L.L., Rivers C.S. (2010). A Hierarchy of Self-Renewing Tumor-Initiating Cell Types in Glioblastoma. Cancer Cell.

[95] Annovazzi L., Mellai M., Schiffer D. (2017). Chemotherapeutic Drugs: DNA Damage and Repair in Glioblastoma. Cancers.

[96] Beier D., Schulz J.B., Beier C.P. (2011). Chemoresistance of glioblastoma cancer stem cells—Much more complex than expected. Mol. Cancer.

[97] Hsieh A., Ellsworth R., Hsieh D. (2011). Hedgehog/GLI1 regulates IGF dependent malignant behaviors in glioma stem cells. J. Cell. Physiol..

[98] Beier D., Röhrl S., Pillai D.R., Schwarz S., Schughart K.L.A., Leukel P., Proescholdt M.A., Brawanski A., Bogdahn U., Trampe-Kieslich A. (2008). Temozolomide Preferentially Depletes Cancer Stem Cells in Glioblastoma. Cancer Res..

[99] Da Ros M., De Gregorio V., Iorio A.L., Giunti L., Guidi M., De Martino M., Genitori L., Sardi I. (2018). Glioblastoma Chemoresistance: The Double Play by Microenvironment and Blood-Brain Barrier. Int. J. Mol. Sci..

[100] Uribe D., Torres Á., Rocha J.D., Niechi I., Oyarzún C., Sobrevia L., Martín R.S., Quezada C. (2017). Multidrug resistance in glioblastoma stem-like cells: Role of the hypoxic microenvironment and adenosine signaling. Mol. Asp. Med..

[101] Fleurence J., Bahri M., Fougeray S., Faraj S., Vermeulen S., Pinault E., Geraldo F., Oliver L., Véziers J., Marquet P. (2019). Impairing temozolomide resistance driven by glioma stem-like cells with adjuvant immunotherapy targeting O-acetyl GD2 ganglioside. Int. J. Cancer.

[102] Kitabayashi T., Dong Y., Furuta T., Sabit H., Jiapaer S., Zhang J., Zhang G., Nakada M. (2018). Dis-Identification of Gsk3β Inhibitor Kenpaullone as a Temozolomide Enhancer Against Glioblastoma. Neuro-Oncology.

[103] Morgan M.A., Canman C.E. (2018). Replication Stress: An Achilles’ Heel of Glioma Cancer Stem–like Cells. Cancer Res..

[104] Todorova P.K., Mukherjee B., Burma S. (2017). MET signaling promotes DNA repair and radiation resistance in glioblastoma stem-like cells. Ann. Transl. Med..

[105] Gherardi E., Birchmeier W., Birchmeier C., Woude G.V. (2012). Targeting MET in cancer: Rationale and progress. Nat. Rev. Cancer.

[106] De Bacco F., Ambrosio D.A., Casanova E., Orzan F., Neggia R., Albano R., Verginelli F., Cominelli M., Poliani P.L., Luraghi P. (2016). MET inhibition overcomes radiation resistance of glioblastoma stem-like cells. EMBO Mol. Med..

[107] Mccord A.M., Jamal M., Williams E.S., Camphausen K., Tofilon P.J. (2009). CD133+ Glioblastoma Stem-like Cells are Radiosensitive with a Defective DNA Damage Response Compared with Established Cell Lines. Clin. Cancer Res..

[108] Severini L.L., Quaglio D., Basili I., Ghirga F., Bufalieri F., Caimano M., Balducci S., Moretti M., Romeo I., Loricchio E. (2019). A Smo/Gli Multitarget Hedgehog Pathway Inhibitor Impairs Tumor Growth. Cancers.

[109] Golán I., De La Fuente L.R., Costoya J.A. (2018). NK Cell-Based Glioblastoma Immunotherapy. Cancers.

[110] Yang D., Sun B., Dai H., Li W., Shi L., Zhang P., Li S., Zhao X. (2019). T cells expressing NKG2D chimeric antigen receptors efficiently eliminate glioblastoma and cancer stem cells. J. Immunother. Cancer.

[111] Neidert M.C., Kowalewski D.J., Wolpert F., Stevanovic S., Rammensee H., Lamszus K., Westphal M., Regli L., Weller M., Eisele G. (2017). P06.05 The natural HLA ligandome of glioblastoma stem-like cells: Antigen discovery for T-cell based immunotherapy. Neuro-Oncology.

[112] Cortez C.E.G., Felix G.V., López E.R., Sotelo J., Canseco M.C., La Cruz V.P.D., Pineda B. (2019). Production and Evaluation of an Avian IgY Immunotoxin against CD133+ for Treatment of Carcinogenic Stem Cells in Malignant Glioma: IgY Immunotoxin for the Treatment of Glioblastoma. J. Oncol..

[113] Wang X., Yang K., Wu Q., Kim L.J.Y., Morton A.R., Gimple R.C., Prager B.C., Shi Y., Zhou W., Bhargava S. (2019). Targeting pyrimidine synthesis accentuates molecular therapy response in glioblastoma stem cells. Sci. Transl. Med..

[114] Ichimura K., Pearson D.M., Kocialkowski S., Bäcklund L.M., Chan R., Jones D.T., Collins V.P. (2009). IDH1 mutations are present in the majority of common adult gliomas but rare in primary glioblastomas. Neuro-Oncology.

[115] Molenaar R.J., Maciejewski J.P., Wilmink J.W., Van Noorden C.J.F. (2018). Wild-type and mutated IDH1/2 enzymes and therapy responses. Oncogene.

[116] Li S., Chou A.P., Chen W., Chen R., Deng Y., Phillips H.S., Selfridge J., Zurayk M., Lou J.J., Everson R.G. (2013). Overexpression of isocitrate dehydrogenase mutant proteins renders glioma cells more sensitive to radiation. Neuro-Oncology.

[117] Satterlee A.B., E Dunn D., Lo D.C., Khagi S., Hingtgen S. (2019). Tumoricidal stem cell therapy enables killing in novel hybrid models of heterogeneous glioblastoma. Neuro-Oncology.

